# Impulse Conduction Increases Mitochondrial Transport in Adult Mammalian Peripheral Nerves *In Vivo*

**DOI:** 10.1371/journal.pbio.1001754

**Published:** 2013-12-31

**Authors:** Marija Sajic, Vincenzo Mastrolia, Chao Yu Lee, Diogo Trigo, Mona Sadeghian, Angelina J. Mosley, Norman A. Gregson, Michael R. Duchen, Kenneth J. Smith

**Affiliations:** 1 Department of Neuroinflammation, University College London, Institute of Neurology, London, United Kingdom; 2 Department of Cell and Developmental Biology, University College London, London, United Kingdom; Stanford University School of Medicine, United States of America

## Abstract

Matching energy supply and demand is critical in the bioenergetic homeostasis of all cells. This is a special problem in neurons where high levels of energy expenditure may occur at sites remote from the cell body, given the remarkable length of axons and enormous variability of impulse activity over time. Positioning mitochondria at areas with high energy requirements is an essential solution to this problem, but it is not known how this is related to impulse conduction *in vivo*. Therefore, to study mitochondrial trafficking along resting and electrically active adult axons *in vivo*, confocal imaging of saphenous nerves in anaesthetised mice was combined with electrical and pharmacological stimulation of myelinated and unmyelinated axons, respectively. We show that low frequency activity induced by electrical stimulation significantly increases anterograde and retrograde mitochondrial traffic in comparison with silent axons. Higher frequency conduction within a physiological range (50 Hz) dramatically further increased anterograde, but not retrograde, mitochondrial traffic, by rapidly increasing the number of mobile mitochondria and gradually increasing their velocity. Similarly, topical application of capsaicin to skin innervated by the saphenous nerve increased mitochondrial traffic in both myelinated and unmyelinated axons. In addition, stationary mitochondria in axons conducting at higher frequency become shorter, thus supplying additional mitochondria to the trafficking population, presumably through enhanced fission. Mitochondria recruited to the mobile population do not accumulate near Nodes of Ranvier, but continue to travel anterogradely. This pattern of mitochondrial redistribution suggests that the peripheral terminals of sensory axons represent sites of particularly high metabolic demand during physiological high frequency conduction. As the majority of mitochondrial biogenesis occurs at the cell body, increased anterograde mitochondrial traffic may represent a mechanism that ensures a uniform increase in mitochondrial density along the length of axons during high impulse load, supporting the increased metabolic demand imposed by sustained conduction.

## Abstract 2

Observations of nerve axons *in vivo* reveal that electrical activity increases the number and speed of transported mitochondria, showing how sudden increases in energy demand may be satisfied.

## Author Summary

As mitochondria are the main power source for most cells, their correct localization is vital for cellular homeostasis. The morphological and functional complexity of neurons, and the unpredictability of energy demand resulting from sustained impulse activity, means that correct mitochondrial function and location is particularly important for these cells. However, it is unclear how mitochondrial transport responds to the wide range of physiological conduction frequencies experienced by an intact nervous system. Here we image mitochondria moving along mouse saphenous nerve axons *in vivo* and observe the effects of sustained trains of impulses, evoked either by electrical stimulation of myelinated axons at physiological frequencies (1 and 50 Hz), or chemical stimulation (cutaneous capsaicin) in unmyelinated axons. We find that the number and speed of mitochondria moving towards the peripheral sensory terminals dramatically increases in active axons. The increased numbers of mobile mitochondria seem to originate from “pinched off” segments of stationary mitochondria (mitochondrial fission), and from recruitment of small stationary mitochondria. The transported mitochondria appear to accumulate in sensory nerve terminals in the skin. This study suggests that mitochondrial fission is involved in rapidly supplying mobile mitochondria to aid their re-distribution to regions of increased metabolic demand along axons.

## Introduction

Axons can be a metre, or more, in length and mitochondria traffic along them to become correctly distributed to fulfil local metabolic requirements [1]. Indeed, defects in mitochondrial transport are associated with a number of diseases affecting long neuronal tracts [2]. The devastating consequence of impaired mitochondrial trafficking is illustrated in the axonal form of Charcot-Marie-Tooth disease, which is caused by selective impairment of mitochondrial transport due to mutations of the mitofusin2 gene [3],[4]. However, studies of dynamic processes such as mitochondrial transport are not feasible in humans and are still very challenging in animal models. Thus, the study of axonal mitochondrial traffic has largely been limited to *in vitro* methods, e.g., employing cultured dorsal root ganglia (DRG), organotypic slice cultures [5], or excised myelinated nerves [6]. However, observations *in vitro* are inevitably limited by inherent constraints. For example, neurites in DRG cultures are not only typically unmyelinated, but they also continue to develop and grow while being examined, so mitochondrial trafficking reflects changes in growth and development as well as the steady state energy requirements of axons. Organotypic slices and excised nerves may contain myelinated axons, but they have no perfused vasculature providing oxygen and nutrients, thus lacking physiological regulatory mechanisms crucial for metabolic adaptation in response to increased demand. Furthermore, excised nerves lack cell bodies and are therefore incipiently undergoing degeneration. To avoid these problems, zebrafish embryos have been used to image mitochondrial trafficking *in vivo* [7], but it is not known whether developing axons in fish reflect the properties of adult mammalian axons. Axonal mitochondria provide the ATP required to support impulse activity, yet almost no information is available about the modulation of axonal mitochondrial traffic by neuronal activity. To overcome the limitations imposed by *in vitro* conditions we have studied mitochondrial traffic *in vivo*, along resting and electrically active adult mammalian axons. Thus, confocal time lapse imaging of mitochondrial movements, distribution, and morphology along saphenous nerve axons in anaesthetised mice was combined with electrical and pharmacological stimulation of myelinated and unmyelinated axons.

## Results

### Impulse Activity Rapidly Increases the Number of Transported Mitochondria

**Figure 1:**
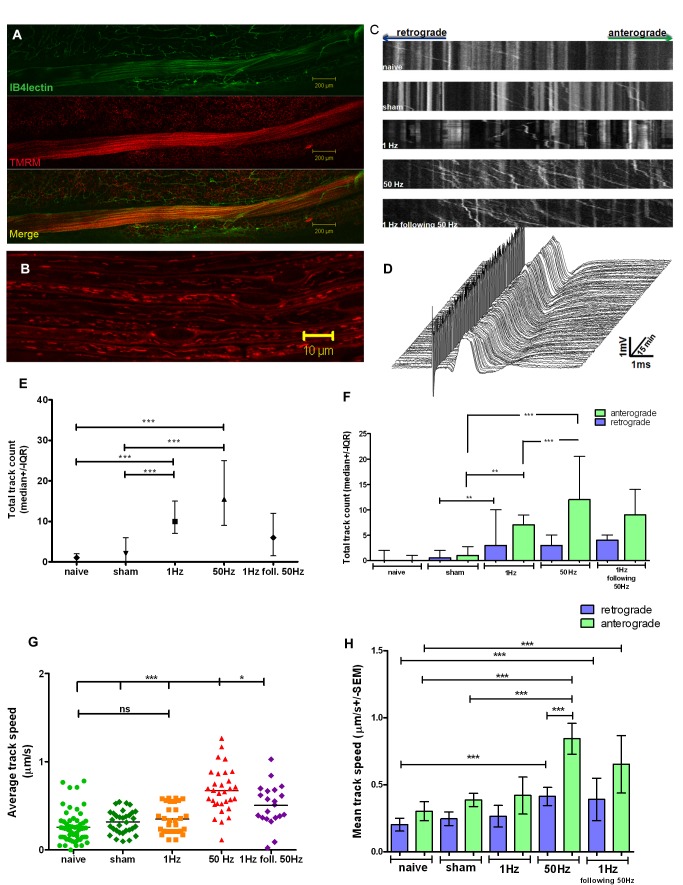
Mitochondrial traffic during impulse conduction. (A) Confocal image of saphenous nerve exposed in an anaesthetised mouse, labelled with IB4-isolectin (green) and TMRM (red). (B) Typical image of saphenous fibres labelled with TMRM. Asterisks denote Schwann cell nuclei. (C) Kymograph analysis of mitochondrial movement in representative axons. (D) CAPs averaged every 30 s during 50 Hz stimulation for 75 min during time-lapse confocal imaging remained similar in form. (E) Impulse conduction significantly increased the number of trafficking mitochondria. (F) Anterograde and retrograde mitochondrial trafficking increased in axons conducting at 1 Hz (*n* = 30 axons) and 50 Hz (*n* = 31 axons) versus naive (*n* = 60 axons) (*p*<0.001) and sham-stimulated animals (*n* = 32 axons) (*p*<0.01): anterograde trafficking is selectively and markedly raised with 50 Hz stimulation (*p*<0.001). (G) Trafficking velocity is significantly higher at 50 Hz than in naive, sham, and 1 Hz-stimulated axons (*p*<0.001), and it decreases upon reducing stimulation to 1 Hz (*n* = 23 axons) (*p*<0.05): anterograde velocity is particularly increased (H). In all groups axons were pulled from three independent experiments (animals). Data that followed normal (Gaussian) distribution are represented as mean ± SEM, whereas data that did not follow normal distribution are represented as median ± IQR. Kruskal-Wallis test with Dunn's multiple comparison test; *p<0.05, **p<0.01, and ***p<0.001.

The mouse (cyan fluorescent protein [CFP^+^] or CFP^−^, see Methods) saphenous nerve was surgically exposed *in situ* taking care not to disturb the vasculature. The mitochondria were labelled by the fluorescent cationic dye tetramethylrhodamine methyl ester (TMRM), which is accumulated by polarised mitochondria. Blood vessels, myelin, perineurium, and the structure (outline) of the axons were labelled using IB4-isolectin, which also labels a subset of IB4^+^ unmyelinated fibres (Figure 1A). Myelinated axons were excited using stimulating electrodes on the saphenous nerve at the groin, and responses monitored at the ankle by an “active” recording electrode (described in detail in Methods). Supramaximal stimuli were delivered at 1 Hz or 50 Hz and the evoked compound action potentials (CAPs) recorded every 30 s. Images were acquired (1.97 s/frame) using a ×63 (oil) warmed objective (Figure 1B; Video S1). Analysis of mitochondrial movement along axons (∼4–6 µm diameter) that crossed the field of view (124 µm) was performed blind using “Difference Tracker” [8] plug-in for ImageJ, and confirmed using ImageJ plug-in “Kymograph” (Figure 1C).

**Figure 2:**
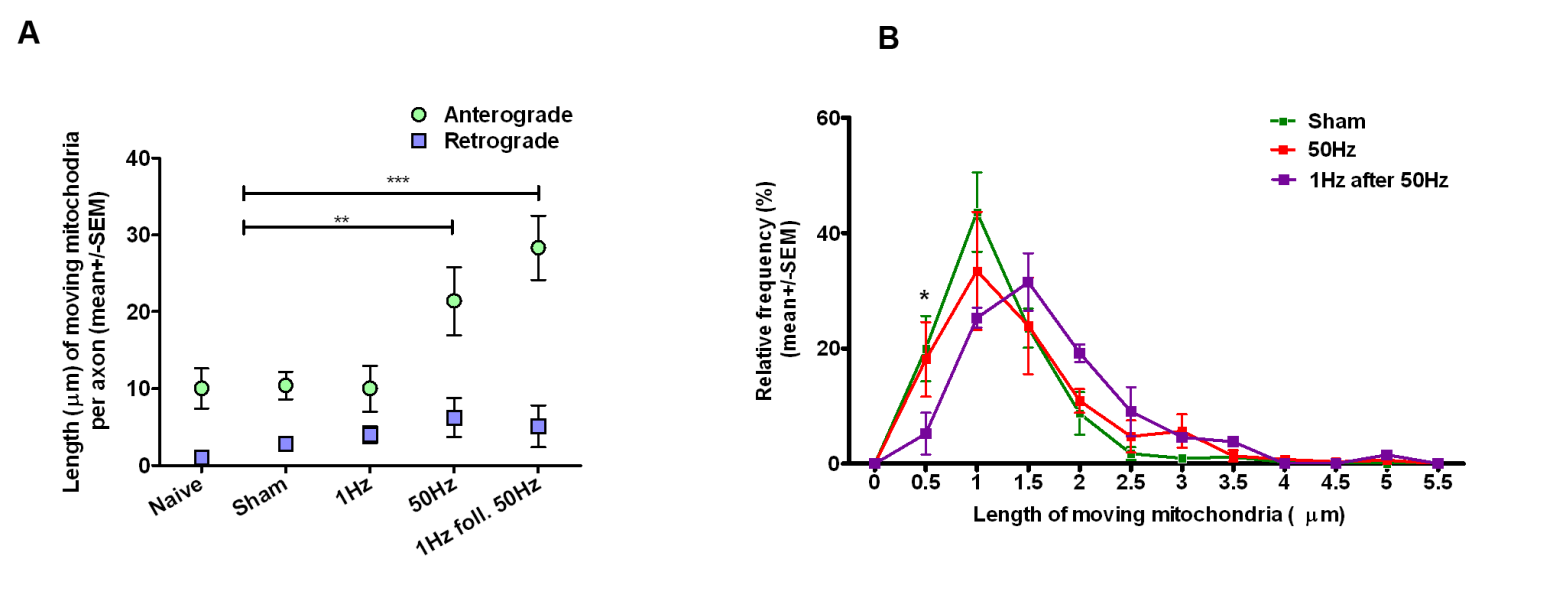
Direction of mitochondrial transport varies depending on frequency of impulse conduction during impulse activity. (A) The total length of mitochondria transported anterogradely (the sum of the length of all mitochondria transported towards the periphery, µm) was significantly greater in axons stimulated at, or after, 50 Hz (*n* = 31) (*p*<0.01; *p*<0.001), than in resting axons, or in axons stimulated at 1 Hz (*n* = 30). (B) The relative frequency of long mobile mitochondria (2.5 µm or longer) was higher in axons conducting at high frequency (50 Hz), or at 1 Hz after 50 Hz.

The CAPs remained unchanged for the entire duration of the stimulation and imaging period (up to 2 h) (Figure 1D), indicating that stimulation at these frequencies was tolerated by the axons *in vivo*. The number of mitochondrial “tracks” (defined as one mitochondrial profile moving continuously for at least 6 consecutive frames [i.e., 11.8 s]) was recorded over 50 frames (i.e., 98.5 s) under the following experimental conditions: naive (*n* = 60 axons from three animals), sham stimulation (*n* = 32 axons from three animals), stimulation at 1 Hz (*n* = 30 axons from three animals), or 50 Hz (*n* = 31 axons from 3 animals), and stimulation at 1 Hz following 50 Hz stimulation (*n* = 23 axons from 2 animals). The overall distribution and morphology of axonal mitochondria in animals prior to stimulation was the same as that in naïve animals. While the majority of mitochondria in unstimulated nerves were stationary, stimulation dramatically increased mitochondrial transport. Thus the median number of moving mitochondria per axon in naïve and sham-stimulated animals was 1 (with inter-quartile range [IQR] 0–2; Video S2) and 2 (IQR 0–6; Video S3), respectively. However, in axons conducting at 1 Hz (Video S4) or 50 Hz (Figure 1E; Videos S1 and S5) the median number of tracks increased significantly to 10 (7–15) (*p*<0.001), and 15.5 (9–25) (*p*<0.001), respectively. The increase at 50 Hz was apparent within the first 10 min of stimulation, when the number of moving mitochondria had already increased to 7 (4–8.5), which was significantly more than in other groups during the same period (*p*<0.01; Figure S1A and S1C). Furthermore, the direction of mitochondrial transport varied with the rate of impulse conduction. While stimulation at 1 Hz increased the number of mitochondria moving in both the anterograde and retrograde directions to a similar extent, higher frequency impulse conduction (50 Hz) predominantly increased the number of mitochondria transported anterogradely (Figure 1F). Furthermore, the total mitochondrial mass moving anterogradely was not balanced by retrograde transport (*p*<0.01, two-way ANOVA), and remained unbalanced even after reducing the frequency of stimulation from 50 Hz to 1 Hz (Figure 2A). In addition, the mobile mitochondria in axons stimulated at 50 Hz were significantly longer than in those stimulated at 1 Hz (*p*<0.001; Figure 2B) such that mobile mitochondria longer than 3 µm were quite common (Videos S1 and S5, arrows), whereas they were rare in less active axons.

### Impulse Activity Gradually Increases the Speed of Mitochondrial Transport

Impulse activity also increased the velocity of moving mitochondria. The average track velocity in axons of naïve animals (0.30 [standard error of the mean (SEM) = 0.02] µm/s), sham-stimulated (0.32 [0.02] µm/s), and 1 Hz-stimulated axons (0.36 [0.03] µm/s) were similar regardless of the direction of movement (*p*<0.05; Figures 1G and S1B), whereas in axons conducting at 50 Hz the average velocity of mitochondrial transport more than doubled to 0.7 (0.04) µm/s (*p*<0.001) (Figure 1H), with anterogradely transported mitochondria moving significantly faster than those transported retrogradely (*p*<0.001) (Figure 1H). Unlike the rapid increase in the number of moving mitochondria following the onset of 50 Hz stimulation (Figure S1C), the velocity increased gradually over 40 min, and returned towards the resting velocity once the stimulation frequency was reduced from 50 Hz to 1 Hz (average velocity reduced to 0.5 [0.06] µm/s within the next 40 min (Figure S1B). These findings suggest that neuronal activity regulates mobilisation of stationary mitochondria and the change in the velocity of mitochondrial transport by distinct mechanisms.

It has been reported that in chick DRG cells in culture, retrogradely moving mitochondria had a lower membrane potential than those transported anterogradely, raising the suggestion that they may be transported to the cell body for degradation [9]. However, in the current study we found no difference in membrane potential between anterogradely and retrogradely transported mitochondria (Figure S1D).

### Electrical Activity at Physiological Frequencies Results in the Accumulation of Mitochondria in Cutaneous Nerve Terminals

**Figure 3:**
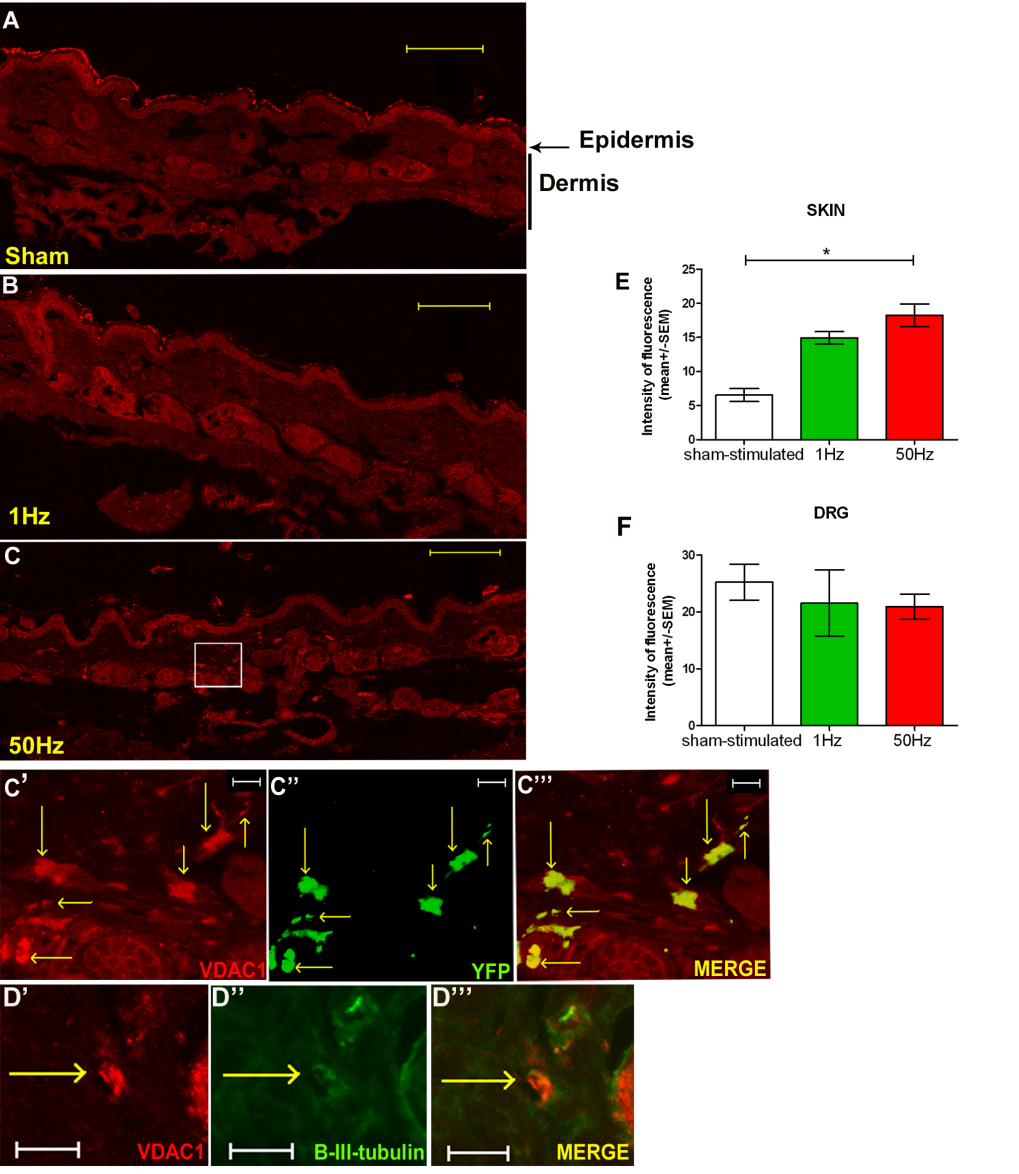
In saphenous nerve axons stimulated at high frequency (50 Hz) mitochondria accumulate at the peripheral sensory terminals. Skin innervated by saphenous nerve was immunohistochemically labelled with VDAC1 (red) and neuron-specific β-III-tubulin (green). In comparison with sham-stimulated animals (A), axons were strongly labelled for VDAC1 in nerves stimulated at 1 Hz (B) and 50 Hz (C). (C′–C′″) Saphenous nerve from a YFP^+^ mouse (shown in low power in C), which expresses YFP (green) in a proportion of fibres, was stimulated with 50 Hz and labelled with VDAC1 (red). (D′–D′″) Saphenous nerve from a YFP^−^ mouse was stimulated with 50 Hz and double labelled with VDAC1 (red) and β-III-tubulin. The two markers were often found to co-localise. (E) Intensity of VDAC1 labelling was significantly higher within cutaneous fibres of saphenous nerve stimulated at 50 Hz (*n* = 5, *p*<0.05) than in sham-stimulated (*n* = 3) or fibres stimulated at 1 Hz (*n* = 3). (F) There was no difference in VDAC1 labelling intensity in DRGs of saphenous nerves between the groups. Scale bars in (A–C) = 100 µm, in (C′–C′″) = 20 µm, in (D′–D′″) = 10 µm.

The disproportionate increase in anterograde mitochondrial transport induced by electrical stimulation raises the possibility that mitochondria may accumulate in the distal portions of axons. To assess this possibility we immunohistochemically labelled the skin innervated by the stimulated saphenous nerve (Figure 3), and the corresponding DRGs, for the mitochondrial marker voltage-dependent anion channel 1, porin (VDAC1) and the neuronal marker β-III-tubulin, following 2 h of either sham-stimulation (*n* = 3), or stimulation at 1 Hz (*n* = 3), or 50 Hz (*n* = 5). Mitochondria were identified in the skin sections (Figure 3A–3C), often within small nerve fibres in the dermal layer of the skin, as assessed by co-localization with yellow fluorescent protein (YFP) transgenically expressed in a proportion of YFP^+^ mice (Figure 3C′–3C′″), and by co-labelling with β-III-tubulin (Figure 3D′–3D′″). These nerve fibres were often strongly labelled for VDAC1 in nerves stimulated at 1 Hz and 50 Hz, but not in sham-stimulated controls. Indeed, image analysis revealed that stimulation at either frequency resulted in over a 2-fold increase in intensity of VDAC1 labelling (Figure 3E), a statistically significant increase in nerves stimulated at 50 Hz. The increase in cutaneous labelling was not matched by an increase in VDAC1 labelling in the corresponding DRGs of the axons (Figure 3F).

### In Adult Axons, Physiological High Frequency Conduction Does Not Recruit Additional Mitochondria to Nodes Of Ranvier

**Figure 4:**
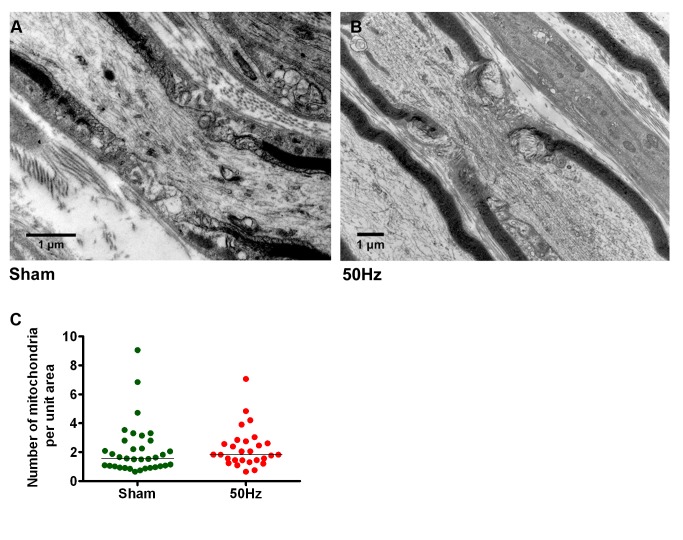
Mitochondria do not accumulate in the vicinity of Nodes of Ranvier. (A and B) Nodes of Ranvier in sham-stimulated (*n* = 35) and axons stimulated at high frequency (50 Hz, *n* = 28) appeared similar upon ultrastructural examination (bar = 1 µm), and (C) possessed similar numbers of mitochondria (*p* = 0.22, Mann-Whitney test).

Next, we examined whether mobilisation of mitochondria affected their distribution along the axons. Nodes of Ranvier are believed to be regions of relatively high energy demand [5] and activity-induced Ca^2+^ elevation [6], so we examined whether mitochondria in stimulated axons accumulated within the vicinity of nodes (i.e., within 15 µm of the nodal gap). Although in axons stimulated at 50 Hz (Video S6) the transport through the nodes intensified (commensurately with the increase in transport along the internode) in comparison with sham-stimulated axons, this mobilisation did not result in accumulation of mitochondria in nodal/paranodal region. We confirmed this finding in a separate set of experiments whereby saphenous nerves were fixed by perfusion following 2 h of either sham- or 50 Hz-stimulation. The number of mitochondria in nodal and paranodal regions was then examined by electron microscopy, which showed no differences between the two groups (Figure 4A and 4B). Similarly, conduction of impulses at 50 Hz for up to 2 h did not result in clustering of mitochondria along the internodes (Figures 4C and S2).

### Impulse Conduction Is Associated with Shorter Stationary Mitochondria

**Figure 5:**
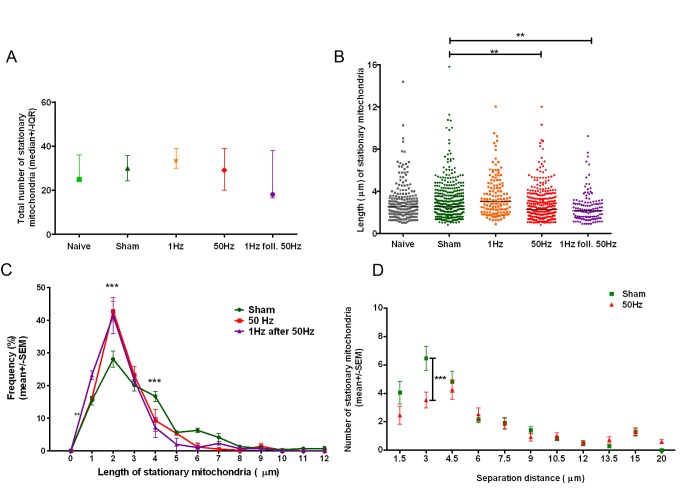
Higher frequency (50 Hz) conduction is associated with shortening of stationary, and an increase in distance between stationary mitochondria. (A) The total number of stationary mitochondrial profiles did not change significantly in response to impulse conduction (*p*>0.05, Kruskal-Wallis test with Dunn's multiple comparison test). (B) Average length of stationary mitochondria was significantly lower in axons conducting impulses at 50 Hz (*n* = 366, *p*<0.01), or at 1 Hz following 50 Hz (*n* = 231, *p*<0.001) than in naive (*n* = 231) or sham-stimulated (*n* = 366) axons. (C). Frequency distribution of length of stationary mitochondria between groups showed a significantly lower number of long mitochondria (i.e., 4 µm) and higher number of short (i.e., 2 µm) in axons conducting at high frequency, than in sham-stimulated axons. (D) The number of mitochondria separated by 1.5 µm (measured between their mid-points) decreased in axons conducting at 50 Hz (*n* = 15 axons, *n* = 288 mitochondria), and was significantly lower for mitochondria separated by 3 µm (*p*<0.001), than in sham-stimulated group (*n* = 13 axons, *n* = 309 mitochondria; three animals per group), i.e., mitochondria were less clustered in stimulated axons. In all groups axons were pulled from three independent experiments (animals).

In order to assess the source of newly mobile mitochondria in electrically active axons, we analysed the number and length of stationary mitochondria in all groups. Surprisingly, the increased number of mobile mitochondria in electrically active axons was not accompanied by a significant decrease in the number of stationary mitochondria (Figure 5A). However, there was a significant decrease in the *length* of stationary mitochondria in axons conducting at 50 Hz and in those conducting at 1 Hz following stimulation at 50 Hz (*p*<0.001) (Figure 5B and 5C). A significant reduction in the length, but not the number, of stationary mitochondria suggests that these mitochondria may have been undergoing fission, although we did not observe this process directly [10]. Similarly, we did not directly observe the moment of recruitment of stationary mitochondria, which could explain a small, but not significant, decrease observed in the number of stationary mitochondria. Therefore, we also analysed the overall distribution of mitochondria along the axon. Indeed, we found that stimulation at 50 Hz resulted in a uniform distribution of mitochondria along the axon/internode such that there were fewer closely spaced mitochondria (i.e., separated by 1.5 and 3 µm) than in other groups (*p*<0.001, two-way ANOVA; Figure 5D). This result suggests that sustained impulse conduction may induce mobilisation of some stationary mitochondria. Interestingly, this recruitment of stationary mitochondria seems to occur preferentially from regions that were densely populated by stationary mitochondria while axons were less active, rather than by random recruitment of stationary mitochondria to the mobile pool. In this way, impulse activity appears to provide a stimulus to increase the number of mobile mitochondria while ensuring an even distribution of mitochondria along the axons, and thereby preventing focal depletion of mitochondria, which would render axons focally vulnerable.

### Pharmacological Stimulation of Unmyelinated Axons Increases Their Mitochondrial Trafficking

**Figure 6:**
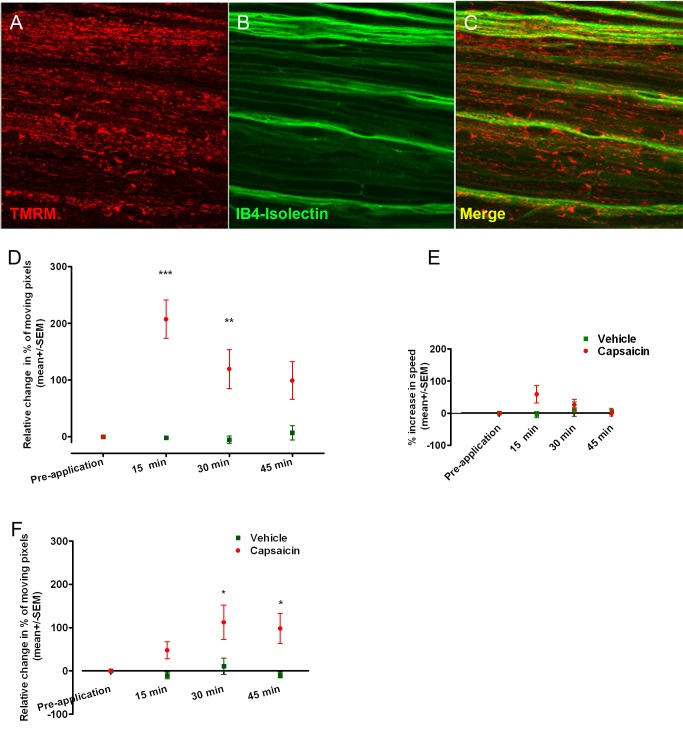
Stimulation of unmyelinated axons by capsaicin increases mitochondrial trafficking. (A–C) Confocal images of saphenous nerve following a single topical application of capsaicin to the skin of the foot, labelled with TMRM (red) and IB4-isolectin (green). A large proportion of unmyelinated fibres were strongly labelled with IB4-isolectin. (D) Within 15 min of capsaicin application (*n* = 27 axons), the percentage of moving mitochondria increased up to 3-fold in comparison with pretreatment, or with vehicle-treated animals (*n* = 10 axons) (*p*<0.001, two-way ANOVA with Bonferroni's post test). Mitochondrial transport was still significantly higher in capsaicin-treated animals after 30 min (*p*<0.01, two-way ANOVA with Bonferroni's post test). (E) The increase in mitochondrial trafficking in myelinated fibres following capsaicin application (*n* = 31) was less dramatic and developed more slowly than in unmyelinated, reaching its peak 30 min post-application (*p*<0.05, two-way ANOVA with Bonferroni's post test), but the increase was more persistent (*p*<0.05, 45 min after capsaicin application, two-way ANOVA with Bonferroni's post test). (F) Capsaicin treatment did not significantly increase the velocity of transported mitochondria in unmyelinated fibres (*p*<0.05, Kruskal-Wallis test with Dunn's multiple comparison test).

In unmyelinated axons, the percentage of moving mitochondria was measured within axons distinguished by labelling with IB4-isolectin^+^ (Figure 6A–6C) before and after the application of capsaicin (3% solution) to the skin on the foot. At steady state, mitochondria visualized by TMRM labelling were much more densely packed in Remak bundles, which contain unmyelinated axons, than in myelinated axons; this latter problem was not overcome by using CFP^+^ mice because these do not express CFP in the mitochondria of unmyelinated axons. However, the image analyses software ignored stationary mitochondria, including those present in the glial cells, thus we identified, imaged, and analysed the moving mitochondria in particular axons at regular intervals before and after applying the vehicle or capsaicin. In vehicle treated preparations mitochondrial movements were consistent across time, but a single application of capsaicin increased the percentage of moving mitochondria in these fibres by more than 200% within 15 min (*p*<0.001; Figure 6D) without an increase in the speed of mitochondrial transport (0.5±0.025 µm/s for vehicle and 0.42±0.1 µm/s for capsaicin) (Figure 6E). The percentage remained significantly increased 30 min after the application in comparison with vehicle-treated preparations (*p*<0.01). Lynn et al. found that action potential firing is promptly evoked by capsaicin and continues for at least 10 min following such capsaicin application [11]. Therefore, the increased mitochondrial trafficking with a peak 15 min after capsaicin application is consistent with the need to satisfy an increase in energy demand resulting from the ionic imbalance created by high frequency conduction in unmyelinated fibres. The exposure to capsaicin also increased the percentage of moving mitochondria in myelinated axons (which were not deliberately stimulated) with a peak at 30 min post-application (*p*<0.05; Figure 6F), although the increase was smaller than in unmyelinated axons. It is possible that the increase in myelinated axons also resulted from activity induced by the application of the capsaicin.

## Discussion

Our findings demonstrate a physiological relationship between impulse activity and mitochondrial trafficking along normal, adult, myelinated, and unmyelinated mammalian peripheral axons *in vivo*. Earlier studies conducted *in vitro* have reported different observations in excised frog nerves [6] and cultured mammalian Purkinje cells in tissue slices [5], where induced activity was associated with a *decrease* in mitochondrial transport, and an accumulation of mitochondria at, or near, nodes of Ranvier. The explanation for the different results is not clear, although there are obvious differences in methodological detail. Zheng et al. applied tetanic stimulation (200 Hz) to excised frog nerves *in vitro*, which is presumably a supra-physiological frequency for this cold-blooded species. Ohno et al. described pharmacologically induced activity in central nervous system Purkinje cells *in vitro*, measured in numbers of calcium transients. The stimulation resulted in an increase from one, to three calcium transients in 200 s, which appears to be an increase in frequency from 0.005 Hz to 0.015 Hz. We have studied impulse activity in mammalian peripheral nervous system (PNS) axons *in vivo* within the physiological range at 0, 1, and 50 Hz (impulse activity at 50 Hz is well within the physiological range for many mammalian axons [12]), suggesting that the relatively high number and speed (average ∼0.7 µm/s and anterograde ∼0.85 µm/s) of moving mitochondria detected with 50 Hz activity may indicate the steady state mitochondrial traffic in sensory axons in awake, active animals. Misgeld and colleagues reported an average speed of mitochondria of 1–1.5 µm/s in intercostal nerve in the triangularis sterni muscle explant [13], but whether the axons were active was not reported. It is possible that the higher speed in the intercostal nerve is due to the presence of motor axons rather than solely sensory axons as in the saphenous nerve, although there is a range of speeds in the saphenous nerve, perhaps reflecting different sensory modalities and their inherently different average levels of impulse activity. To our knowledge, the link between molecular motors (e.g., kinesin and dynein), which carry mitochondria along the microtubules, and impulse conduction is not known. The previous studies into mechanisms of regulation of mitochondrial traffic studied growing, or damaged, axons, and therefore tended to detect and examine docking, rather than the mechanisms of increases in number of moving mitochondria or their speed.

In contrast to descriptions of activity-dependent nodal accumulation of mitochondria *in vitro* [5],[6], we found no such evidence *in vivo*. Although it seems reasonable to expect a greater increase in energy demand at nodes of Ranvier during sustained impulse activity, sodium entry to the axon during impulse activity is a passive process and the energy-demanding act of sodium extrusion via the Na^+^/K^+^ ATPase appears to be accomplished along the internode [14]. The absence of nodal recruitment of mitochondria in response to physiological levels of impulse activity is therefore unsurprising. Also, one might expect that the mitochondria in the mouse nerves are already optimally positioned along the axons by physiological impulse activity because of prior life experience, and so re-positioning in response to the applied impulse load is unnecessary. The nodal accumulation observed in the frog nerves may have been related to the unusually high stimulus rate of 200 Hz applied to this amphibian species, or to the fact that amphibian nodes can have different biophysical properties to mammalian nodes, e.g., with regard to the distribution of potassium channels [15], and perhaps to the distribution of the Na^+^/K^+^ ATPase.

The large number of mitochondria normally found at sensory axon terminals [16],[17] indicates that the terminals have a high metabolic demand, and the surge of anterograde transport we have observed will help to satisfy this demand. Indeed, we demonstrated increased labelling for the mitochondrial marker, VDAC1, in the dermal terminals of saphenous nerves conducting at 50 Hz, suggesting that the sensory endings are one of the destinations of the anterogradely transported mitochondria. A recent study found that just one sensory neuron can branch to make contact with around 200 hair follicles or Merkel cells [18], and individual neuronal arbors in hairy skin may have around 1,000 branch points, and a total cutaneous axon length of up to one metre. In this case, providing even a small increase in mitochondrial density to all the terminals of one axon would require a substantial increase in anterograde mitochondrial trafficking in the parent axon. Indeed, failure to satisfy such potential demand for mitochondria in sensory terminals in disease states may explain the dying-back neuropathies, such as Charcot-Marie-Tooth (CMT) or diabetic neuropathy: indeed, CMT is associated with an impairment of mitochondrial trafficking due to mutation of the mitofusin2 gene.

The destiny of mitochondria accumulated within peripheral terminals, following cessation of high frequency impulse conduction, is still unclear. If the purpose of transport is to deliver functional mitochondria to the target destination (e.g., sensory terminal), then it seems counterproductive for the neuron to degrade these, presumably healthy, mitochondria in the terminals once the period of increased need is ceased. Previous studies suggested that mitochondrial degradation and autophagy is indeed initiated at the axonal terminal of cultured DRG neurons, and that the autophagosomes are then transported retrogradely towards the cell body, becoming increasingly acidified along the way [19].

In our study, the increase in the mobile cohort of mitochondria appears to be composed of (a) static mitochondria that become mobile, particularly from regions densely populated by small mitochondria, and (b) portions of mitochondria that are pinched off the static population by fission. A possibly comparable phenomenon of depolarisation-induced fission has been observed in the dendritic spines of cultured hippocampal neurons [20]. The mechanism(s) by which fission contributes to neuroprotection are not established. Our study suggests a hitherto unsuspected role of fission in providing a rapid supply of mobile mitochondria to axons that are required promptly to conduct impulses at high frequencies over relatively long periods.

Our observations indicate a mechanism by which axons solve the problem of how to match mitochondrial density and distribution to the changing energy demands imposed by a variable impulse load. It would be wasteful to provide more mitochondria than necessary, and yet dangerous to provide too few, so a mechanism to maintain dynamic balance is necessary. A sustained increase in impulse load (e.g., resulting from an injury that causes a shift in body stance) requires an increase in mitochondrial mass, but this has to be achieved promptly and to be evenly distributed along an axon that may be a metre long, even in a moderately sized body such as a human. The conundrum appears to be solved by a mechanism where the longest mitochondria stay anchored (which ensures that no regions are deprived of mitochondria), but “pinch off” daughter mitochondria by fission, adding them to the anterograde traffic so that they increase mitochondrial density evenly along the axon, building an increased mitochondrial mass from the terminals backwards as the demand is satisfied. New mitochondria are presumably added to the proximal axon at the cell body to maintain the proximal population, and to sustain a raised mitochondrial mass suitable for the new impulse load. Aside from responding to changes in impulse load, the effect of impulse activity on mitochondrial trafficking also provides an effective mechanism to ensure that mitochondria are replenished more rapidly along axons that have a higher impulse activity. It seems likely that mitochondria with high rates of oxidative phosphorylation in very active axons will require earlier replacement, and in this case the mechanism would ensure that mitochondria cycle rapidly through very active axons, but linger longer in more quiescent axons.

## Materials and Methods

### Animals

All animal experiments were carried out according to the 1986 Animals (Scientific Procedures) Act, UK, and were approved by the institutional ethics committee. Mice used in this study were male, 8–12 weeks old, and either Thy-1-YFP-16 positive or negative littermates (Jackson laboratories strain designation: B6.Cg-Tg [Thy1-YFP]16Jrs/J), Thy1-CFP-S positive or negative littermates (Jackson laboratories strain designation: B6;CB-Tg [Thy1-CFP/COX8A)S2Lich/J], or C57Bl/6 mice. YFP mice were used to assess any axonal damage caused by surgery or prolonged imaging as the YFP expressed in a proportion of axons reveals altered axonal structure. CFP-S mice were used to distinguish axonal from Schwann (and other) cell mitochondria, as the fluorescent probe is expressed selectively in axons, affecting approximately 40%–60% of them. The CFP labelled mitochondria fluoresce blue regardless of their membrane potential, and so depolarised (probably damaged) mitochondria are not distinguished from their polarised (probably healthy) counterparts using only this label. Therefore, the membrane potential was revealed by the topical application of the cationic, potentiometric dye TMRM (Invitrogen), which partitions into polarised mitochondria. Any experiments (animals) in which axons showed any morphological abnormalities such as physical damage, loss of cylindrical shape of axons, etc., or in which CFP mitochondria were not co-labelled with TMRM marks (polarised mitochondria), either before or after time-lapse imaging were considered to be possibly damaged and thus were excluded from analysis.

### Surgical Procedure, Electrophysiology, and Imaging

Mice were terminally anaesthetised (urethane; 1.25 g/kg), the skin was opened at the thigh, and the connective tissue carefully removed. Approximately 1 cm of the left saphenous nerve was exposed in the middle of the thigh and desheathed, taking care to avoid any damage to the nerve fibres or the vasculature. The nerve was labelled with TMRM (1 µM solution in sterile saline; pH = 7), by soaking cotton balls with the TMRM solution and placing them over the entire exposed part of the nerve for 45 min. Additionally, the nerve was labelled with Griffonia isolectin IB4 (Sigma; 1∶20 in sterile saline) in the same way, for 10 min. Our preliminary experiments showed that all axonal mitochondria are labelled in this way, including those several millimetres (e.g., 2–3 mm) distal and proximal to the desheathed site. The excess dyes were removed by repeated washing with sterile saline. For electrophysiological experiments, the saphenous nerve was additionally exposed in the groin by cutting the skin and removing the fat tissue to create a well with the nerve at the bottom. A piece of Teflon tape approximately 2 cm×0.5 cm was carefully placed under the nerve in order to isolate the nerve electrically from the surrounding muscle tissue. A pair of stimulating platinum electrodes held by micromanipulators was carefully placed under the saphenous nerve, resting on the Teflon tape. The well was then filled with mineral oil to prevent tissue drying. The active recording electrode was placed directly next to the exposed distal part of the saphenous nerve at the ankle. A reference needle electrode was inserted in the fifth toe, and a ground electrode placed in the abdominal muscle. Once the electrodes were positioned the animal was transferred to the customised stage of a confocal microscope (LSM Pascal 5.0, Zeiss). A single supramaximal stimulus (between 0.8 and 4.8 V, 50 µs) was delivered at a frequency of either 1 Hz or 50 Hz, or no stimuli were delivered (sham-stimulated). Time-lapse or single images of the middle portion of the exposed nerve in the thigh were acquired at 1.97 s per frame using LSM Pascal 5.0 software (Zeiss) and Zeiss Appochromat Plan ×63 (oil) warmed (37°C) objective (NA 1.4). Antidromic sensory compound action potentials (SCAPs) were averaged (*n* = 10) and stored by computer (Sigma 60, Nicolette Technologies) every 30 s concurrently with real time imaging. The rectal temperature was continuously monitored, and maintained at 36°C by an underlying heating mat. We examined five separate groups of animals. For the “naïve” group (*n* = 3 animals) the saphenous nerve was exposed and labelled, but no other manipulations were performed prior to confocal imaging. In the “sham-stimulated” group (*n* = 3) the saphenous nerve was surgically exposed, the electrodes positioned and the equipment turned on, but no electrical stimuli were applied. Two other sets of three animals in the “1 Hz” group, and “50 Hz” group underwent all the same procedures as the “sham-stimulated” group, but stimuli were delivered at 1 Hz or 50 Hz from the start of the experiment. In two additional animals stimulated at “50 Hz” group, the stimulation frequency was reduced to 1 Hz after 60 min of stimulation at 50 Hz, thus creating a “1 Hz following 50 Hz” group.

### Histology and Electron Microscopy

After *in vivo* imaging and/or electrophysiological stimulation, the nerves were fixed by immersion in paraformaldehyde (for immunohistological studies) or glutaraldehyde (for EM studies) for at least 24 h and the central 5 mm length of each fixed nerve, corresponding to the imaging site, was excised. The nerves were washed (3×10 min; 0.1 M PO_4_ buffer; pH 7.4), post-fixed in osmium tetroxide (1.5%; 1 h) and washed again (3×10 min; 0.1 M PO_4_ buffer; pH 7.4). The nerves were dehydrated in an ascending series of alcohols and passed via propylene oxide into resin (TAAB) and embedded in Beem capsules for longitudinal sections, and polymerized at 60°C for 48 h. Ultrathin sections from selected blocks were subsequently examined in an electron microscope (JEM-1010 Electron Microscope, JEOL).

### Immunohistochemistry

To assess the distribution of mitochondria distal to the imaging site following electrical stimulation, sections through the skin of the dorsum of the foot innervated by the left saphenous nerve was double-labelled with the mitochondrial marker VDAC1 and the neuronal marker β-III-tubulin. The fixed skin was dissected and embedded in optimal cutting temperature (OCT) medium (VWR) and frozen at −20°C before cryo-sectioning. The 10-µm-thick cryostat sections were cut and mounted on glass slides and dried at room temperature overnight before being stored at −20°C. Before immunohistochemical labelling, the slides were rehydrated in 0.1 M PBS solution (3×5 min). Antigen retrieval was performed using 10% citric acid (Dako) for 40 min at 40°C. After washing (3×5 min) in 0.1 M PBS containing 0.1% Triton X-100 (PBS-T), slides were incubated in the appropriate serum blocking solutions for 1 h. Primary antibodies polyclonal rabbit anti-VDAC1 (1∶600) and monoclonal mouse anti neuronal β-III tubulin (1∶1,000) were diluted in blocking solution and applied overnight at 4°C. After subsequent washes with PBS-T solution (3×5 min), species-specific secondary antibodies corresponding to the primary antibodies, conjugated to AlexaFluor 546, or 647, were applied for 1 h at room temperature, slides were washed (3×5 min) in PBS-T and mounted using 4′,6-diamidino-2-phenylindole (DAPI) fluorescence mounting medium (Vector Laboratories). All sections were immunolabelled at the same session. For image acquisition, the confocal microscope (LSM 5 Pascal Exciter; Zeiss) was used. Fluorescence emission was recorded through a 10×/0.3 and PLAN-APOCHROMAT 63×/1.4 Oil Ph3 (Zeiss) objective lenses. All images were acquired with following settings: beam-splitter HFT 488/543; filter BP 505–570, argon laser irradiation at 488 nm and filter LP 585 helium neon laser irradiation at 543 nm. Care was taken to not saturate the intensity of pixels during image acquisition. The immunolabelling and image acquisition were performed in a blinded fashion.

### Image Analysis

The number, direction, and velocity of moving, TMRM positive, mitochondria in the time-lapse videos were analysed using Image J. All analyses were performed on the red channel (i.e., the TMRM labelling) as the signal to noise ratio in this channel was superior to that obtained by imaging CFP. However, the CFP labelling was used to confirm that the analysed particles were indeed axonal, and not Schwann cell, mitochondria (Figure S3). Internodal segments of myelinated axons with calibre between 4 and 6 µm, were first straightened using the Straighten plug-in (http://rsbweb.nih.gov/ij/plugins/straighten.html) for ImageJ version 1.43 software (NIH, rsb.info.nih.gov/ij/). Straightened axons were processed using the Difference Tracker plug-in (http://www.bioinformatics.bbsrc.ac.uk/projects/difference_tracker/) for ImageJ as previously described [8],[21], and fluorescent particles with a lower cut off of five pixels moving for at least six consecutive frames were considered to be moving mitochondria. The lower cut off was determined by examination of the size of the smallest moving CFP and TMRM positive particles. The number of moving mitochondria, and their direction and velocity were directly derived from the results table generated by Mass particle Tracker part of the Difference Tracker plug-in. The results were confirmed by manually tracking mitochondria in 21 randomly chosen axons by a blinded examiner. Kymographs of mitochondrial movement were generated in ImageJ directly from straightened time-lapses. Data derived by manual analyses and using Kymograph plug-in were consistent with those obtained using Difference Tracker.

The length of mobile mitochondria was determined manually by a blinded examiner (VM). First, all axons (124 µm in length) were divided into three areas to ensure that all moving mitochondria were observed and measured. Each mitochondrion observed to be moving for six or more consecutive frames within these areas was measured using the measuring tools in LSM Image Browser software. As mitochondria in our experiments invariably measured to be ∼0.5 µm in width, and only the length varied, the length was used as a measure of mitochondrial size throughout. Length of stationary mitochondria was analysed using the ImageJ Analyse Particle plug-in. First, time-lapse images of axons were straightened using the Straighten plug-in. Background was reduced using intensity subtraction (the radius of the rolling ball was set to 5) in ImageJ and the time lapse was converted into a binary form. Using the drawing tool, all the moving particles were eliminated. The dimensions of remaining stationary particles were analysed using Analyse Particle plug-in for ImageJ, whereby the Feret's Diameter value, was converted into length in micrometres.

The distribution of stationary axonal mitochondria and the distance between them (separation distance) was manually and blindly determined from timed sequences, from randomly selected axons. Suitable axons from naïve animals (*n* = 3 animals, *n* = 13 axons, and total of 252 mitochondria), sham-stimulated (*n* = 3 animals, *n* = 13 axons, and total of 309 mitochondria), and 50 Hz-stimulated (*n* = 3 animals, *n* = 15 axons, and total of 288 mitochondria) were identified in the first, middle, and end frames in ImageJ, straightened if necessary, and stored as individual 8-bit TIFF files. If required, the brightness and contrast were adjusted. In Photoshop, the three axonal images were mounted as a photomontage, aligned, and mitochondria found at the same position in all three frames identified. Using ImageJ the x co-ordinate at the approximate mid-point of each identified stationary mitochondrion was recorded and used as the reference point. The position of other axonal features, i.e., nodes of Ranvier, Schwann cell nuclei, and the axonal diameter, were also recorded, and the distance between the stationary mitochondria and their distance from the node (*n* = 3 animals, *n* = 12 axons, 241 mitochondria for naïve; *n* = 3 animals, *n* = 17 axons, *n* = 204 mitochondria for sham, and *n* = 3 animals, *n* = 15 axons, *n* = 159 mitochondria for 50 Hz) were calculated.

Mitochondrial potential was determined blindly by selecting each moving mitochondrion and its surrounding axoplasm using polygonal selection tool in ImageJ, in randomly selected axons from sham-stimulated (*n* = 3 animals, *n* = 15 axons) or axons conducting at 50 Hz (*n* = 3 animals, *n* = 14 axons). The intensity of TMRM fluorescence across the selections was measured and a ratio between the standard deviation (SD) and mean of the fluorescence intensity was used as a measure of mitochondrial membrane potential [22].

For analyses of mitochondrial distribution in the skin, the relative fluorescence intensity of VDAC1 labelling in the dermal layer of the skin was normalized to the corresponding background intensity and accessed by densitometric analysis in ImageJ software, in a blinded fashion.

### Data Analysis

Prior to all statistical analyses, the data were tested for normality of distribution using D'Agostino and Pearson normality test. Kruskal-Wallis test with Dunn's multiple comparison post-test was used to calculate differences between total track counts and the velocity of mitochondrial transport. Comparison between numbers of mobile and stationary mitochondria of different lengths, differences in separation distances between stationary mitochondria, differences in proportion of mobile mitochondria at nodes/paranodes versus internodes, differences in proportion mitochondrial potential, and differences in proportions of moving mitochondria following capsaicin or vehicle application were all performed using two-way ANOVA with Bonferroni's multiple comparison tests. The numbers of mitochondria in nodal/paranodal regions from electron microscopy images were compared using Mann Whitney test. Differences in VDAC1 labelling intensity in the dermal layer of the skin were calculated using one was ANOVA with Tukey post-test. Parametric data are presented as mean ± SEM, whereas non-parametric, or data that did not follow a normal distribution, are presented as median ± IQR.

## Supporting Information

Figure S1 **Impulse conduction increases the number of transported mitochondria rapidly after the onset of stimulation, and the speed of mitochondrial trafficking gradually, after the onset of stimulation.** No differences in mitochondrial membrane potential between mitochondria travelling in opposite directions. (A) and (C). Within 10 min of conduction at 50 Hz (*n* = 31 axon), the number of mobile mitochondria significantly increases in comparison with sham-stimulated axons (*n* = 32) or naive axons (*n* = 60), (*p*<0.01, Kruskal-Wallis test with Dunn's multiple comparison test). (B). The impulse conduction at 50 Hz, but not 1 Hz, increased the velocity of mitochondrial transport, but this increase in velocity occurred gradually within 40 min and also slowly decreased following change of stimulation from 50 Hz to 1 Hz. (D) Mitochondrial membrane potential of anterogradely (*n* = 22 axons) and retrogradely (*n* = 30 axons) transported mitochondria were not significantly different.
(TIF)

Figure S2 **Mitochondrial distribution does not change.** No accumulation of mitochondria was detected in the nodal/paranodal region in stimulated axons (*n* = 159 mitochondria from 15 axons for 50 Hz stimulation, *n* = 241 from 12 axons for the naive group, and *n* = 204 from 17 axons for the sham-stimulated group; from 3 animals in each group).
(TIF)

Figure S3 **Mitochondrial labelling with TMRM in a CFP-S mouse saphenous nerve.** The overwhelming majority of mitochondria in healthy axons is labelled with both CFP (blue) and TMRM (red), appearing white. Schwann cell mitochondria can be easily distinguished from axonal as the Schwann cell mitochondria are only labelled with TMRM (red arrows).
(TIF)

Video S1 **Time-lapse video showing mitochondrial trafficking in axons conducting at 50 Hz** ***in vivo*** **.** Mouse saphenous nerve was de-sheathed, the mitochondria labelled with TMRM (red), and stimulated proximally at 50 Hz: conducted CAPs were recorded distally to the imaging site. Many mitochondria are present moving in either direction. Some very long mobile mitochondria are also observed (tracked by blue arrow). A node of Ranvier can be seen in the lowermost axon, but no accumulation of mitochondria near the node is apparent (the foot is to the right). Note: Please use Internet Explorer to download; some browsers seem to distort the video and dramatically reduce their quality. Videos may be best viewed in Windows Media Player.
(AVI)

Video S2 **Time-lapse video showing mitochondrial trafficking in resting axons of a naïve animal** ***in vivo*** **.** Mouse saphenous nerve was de-sheathed and the mitochondria labelled with TMRM (red), but no deliberate stimulation occurred. Most mitochondria appear stationary, but some short mitochondria are mobile, moving either towards the foot (right) or proximally. Note: Please use Internet Explorer to download; some browsers seem to distort the video and dramatically reduce their quality. Videos may be viewed in Windows Media Player.
(AVI)

Video S3 **Time-lapse video showing mitochondrial trafficking in sham-stimulated axons** ***in vivo*** **.** Mouse saphenous nerve was de-sheathed and the mitochondria labelled with TMRM (red), and stimulating and recording electrodes were positioned proximally and distally to the imaging site, as described in Methods. The electrophysiological equipment was turned on, but no stimuli were delivered. Most mitochondria appear stationary, but some short mitochondria are mobile, moving either towards the foot (right) or proximally. A node of Ranvier can be seen in the uppermost axon. Note: Please use Internet Explorer to download; some browsers seem to distort the video and dramatically reduce their quality. Videos may be best viewed in Windows Media Player.
(AVI)

Video S4 **Time-lapse video showing mitochondrial trafficking in axons stimulated at 1 Hz** ***in vivo*** **.** Mouse saphenous nerve was de-sheathed, the mitochondria labelled with TMRM (red), and stimulated proximally at 50 Hz: conducted CAPs were recorded distally to the imaging site. Many mitochondria are present moving either towards the foot (right) or proximally. Note: Please use Internet Explorer to download; some browsers seem to distort the video and dramatically reduce their quality. Videos may be best viewed in Windows Media Player.
(AVI)

Video S5 **Time-lapse video showing mitochondrial trafficking in axons stimulated at 50 Hz, including a particularly long retrogradely moving mitochondrion** ***in vivo*** **.** Mouse saphenous nerve was de-sheathed, the mitochondria labelled with TMRM (red), and stimulated proximally at 50 Hz: conducted CAPs were recorded distally to the imaging site. Many mitochondria are present moving in either direction. Some very long mobile mitochondria are also observed (one moving retrogradely is tracked by blue arrow). Two examples of unmyelinated axons with densely packed mitochondria are visible (*). A node of Ranvier can be seen in the second axon from the top, but no accumulation of mitochondria near the node is apparent (the foot is to the right). Note: Please use Internet Explorer to download; some browsers seem to distort the video and dramatically reduce their quality. Videos may be best viewed in Windows Media Player.
(AVI)

Video S6 **Time-lapse video showing mitochondrial trafficking through a Node of Ranvier in an axon stimulated at 50 Hz** ***in vivo*** **.** Mouse saphenous nerve was de-sheathed, the mitochondria labelled with TMRM (red), and stimulated proximally at 50 Hz: conducted CAPs were recorded distally to the imaging site. The node is apparent due to labelling of the many mitochondria present within the Schwann cell at the node. No mitochondria appear to accumulate at the node (nor do they hesitate at this site; the foot is to the right). Note: Please use Internet Explorer to download; some browsers seem to distort the video and dramatically reduce their quality. Videos may be best viewed in Windows Media Player.
(AVI)

## Abbreviations

CAP: compound action potential
CFP: cyan fluorescent protein
DRG: dorsal root ganglion
IQR: inter-quartile range
SEM: standard error of the mean
TMRM: tetramethylrhodamine methyl ester
VDAC1: voltage-dependant anion channel 1
YFP: yellow fluorescent protein
