# Supplementary figures and images for: Impulse Conduction Increases Mitochondrial Transport in Adult Mammalian Peripheral Nerves In Vivo
Source: PLoS Biol. 2013 Dec 31;11(12):e1001754. doi: 10.1371/journal.pbio.1001754 (PMC3876979; doi:10.1371/journal.pbio.1001754)

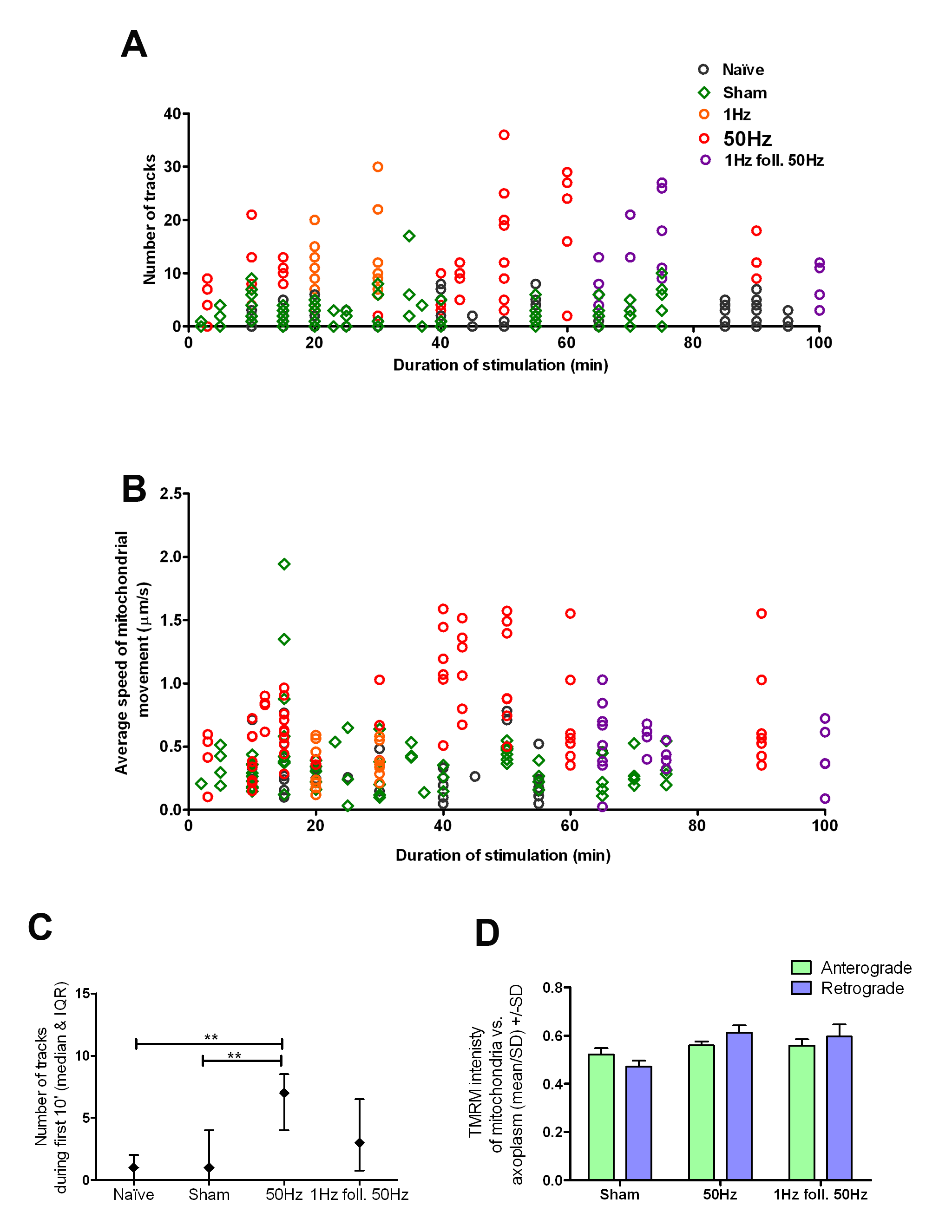

Supplement: Figure S1 — Impulse conduction increases the number of transported mitochondria rapidly after the onset of stimulation, and the speed of mitochondrial trafficking gradually, after the onset of stimulation. No differences in mitochondrial membrane potential between mitochondria travelling in opposite directions. (A) and (C). Within 10 min of conduction at 50 Hz (n = 31 axon), the number of mobile mitochondria significantly increases in comparison with sham-stimulated axons (n = 32) or naive axons (n = 60), (p<0.01, Kruskal-Wallis test with Dunn's multiple comparison test). (B). The impulse conduction at 50 Hz, but not 1 Hz, increased the velocity of mitochondrial transport, but this increase in velocity occurred gradually within 40 min and also slowly decreased following change of stimulation from 50 Hz to 1 Hz. (D) Mitochondrial membrane potential of anterogradely (n = 22 axons) and retrogradely (n = 30 axons) transported mitochondria were not significantly different. (TIF) [file pbio.1001754.s001.tif]

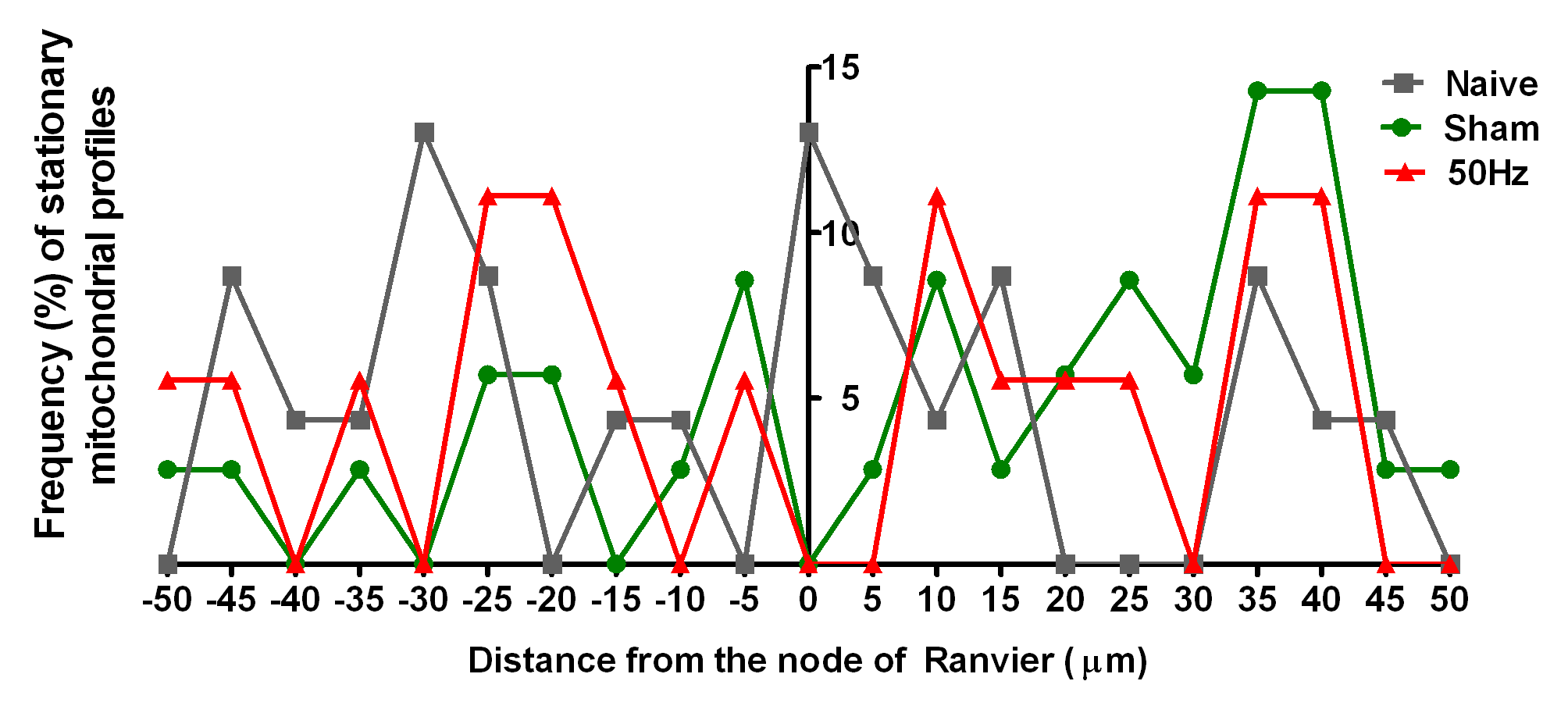

Supplement: Figure S2 — Mitochondrial distribution does not change. No accumulation of mitochondria was detected in the nodal/paranodal region in stimulated axons (n = 159 mitochondria from 15 axons for 50 Hz stimulation, n = 241 from 12 axons for the naive group, and n = 204 from 17 axons for the sham-stimulated group; from 3 animals in each group). (TIF) [file pbio.1001754.s002.tif]

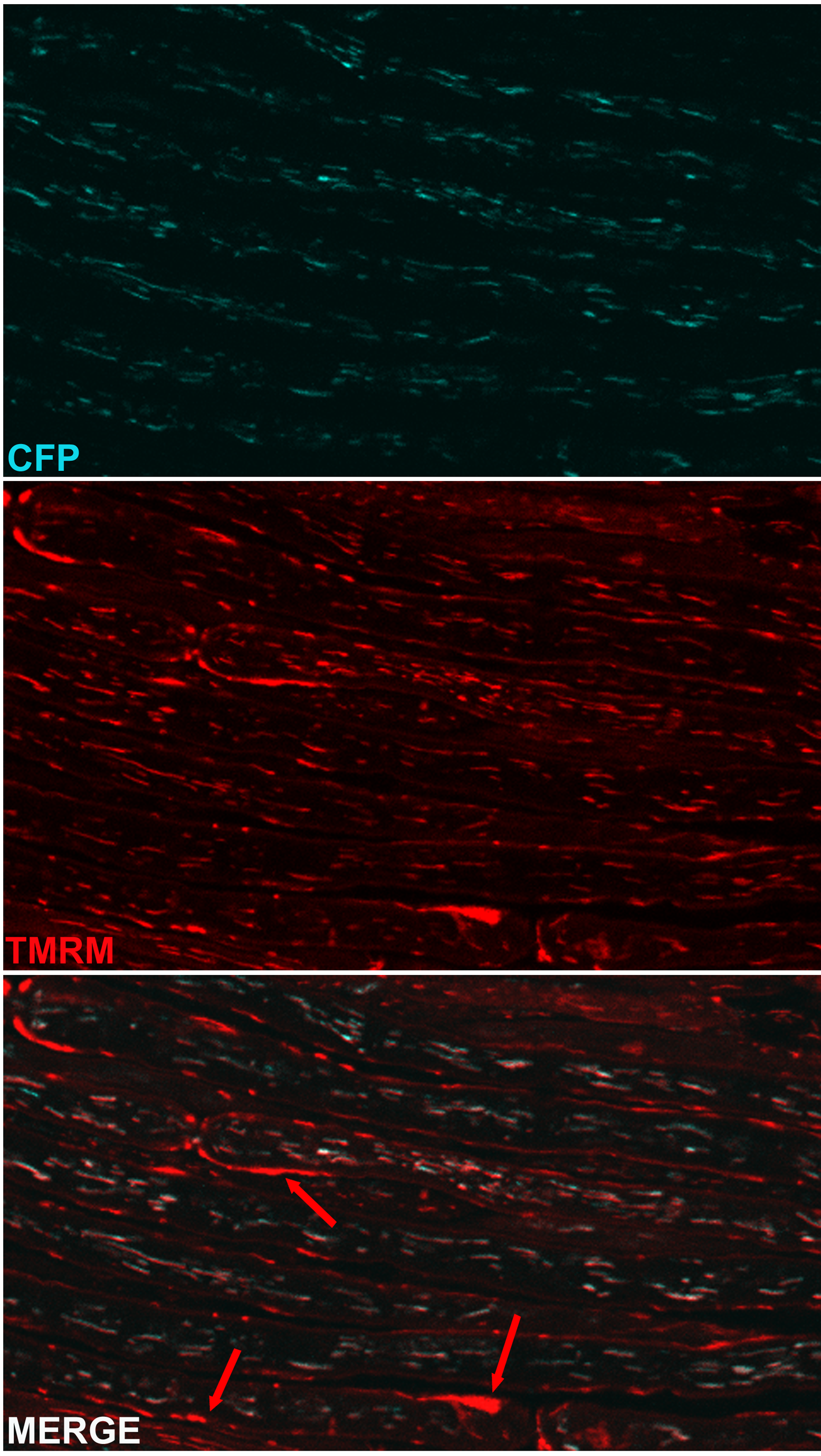

Supplement: Figure S3 — Mitochondrial labelling with TMRM in a CFP-S mouse saphenous nerve. The overwhelming majority of mitochondria in healthy axons is labelled with both CFP (blue) and TMRM (red), appearing white. Schwann cell mitochondria can be easily distinguished from axonal as the Schwann cell mitochondria are only labelled with TMRM (red arrows). (TIF) [file pbio.1001754.s003.tif]
